# On the Influence of Soret and Dufour Effects on MHD Free Convective Heat and Mass Transfer Flow over a Vertical Channel with Constant Suction and Viscous Dissipation

**DOI:** 10.1155/2014/639159

**Published:** 2014-10-28

**Authors:** Ime Jimmy Uwanta, Halima Usman

**Affiliations:** Department of Mathematics, Usmanu Danfodiyo University, PMB 2346, Sokoto State, Nigeria

## Abstract

The present paper investigates the combined effects of Soret and Dufour on free convective heat and mass transfer on the unsteady one-dimensional boundary layer flow over a vertical channel in the presence of viscous dissipation and constant suction. The governing partial differential equations are solved numerically using the implicit Crank-Nicolson method. The velocity, temperature, and concentration distributions are discussed numerically and presented through graphs. Numerical values of the skin-friction coefficient, Nusselt number, and Sherwood number at the plate are discussed numerically for various values of physical parameters and are presented through tables. It has been observed that the velocity and temperature increase with the increase in the viscous dissipation parameter and Dufour number, while an increase in Soret number causes a reduction in temperature and a rise in the velocity and concentration.

## 1. Introduction

The phenomenon of coupled heat and mass transfer by free convection in a fluid saturated porous medium occurs in many engineering and technological and manufacturing industries such as hydrology, geosciences, electronic devices cooled by fans, geothermal energy utilization, petroleum reservoirs, and design of steel rolling and nuclear power plants. A comprehensive account of the available information is provided in the recent books Neild and Bejan [1] and Ingham and Pop [2].

In recent years, considerable attention has been devoted to study the MHD flows of heat and mass transfer because of the applications in geophysics, aeronautics, and chemical engineering. Palani and Srikanth [3] studied the MHD flow of an electrically conducting fluid over a semi-infinite vertical plate under the influence of the transversely applied magnetic field. Makinde [4] investigated the MHD boundary layer flow with heat and mass transfer over a moving vertical plate in the presence of magnetic field and convective heat exchange at the surface. Additionally, Duwairi [5] analyzed viscous and joule-heating effects on forced convection flow from radiate isothermal surfaces. The effect of viscous dissipation is usually characterized by the Eckert number and has played a very important role in geophysical flow and in nuclear engineering that was studied by Alim et al. [6]. It also plays an important role in free convection in various processes on large scales or for large planets. The effects of suction on boundary layer flow also have greater influence over the engineering application and have been widely investigated by numerous researchers. Various authors have studied the effects of viscous dissipation and constant suction in different surface geometries. Uwanta [7] studied the effects of chemical reaction and radiation on heat and mass transfer past a semi-infinite vertical porous plate with constant mass flux and dissipation. Mansour et al. [8] described the influence of chemical reaction and viscous dissipation on MHD natural convection flow. The effect of chemical reaction and heat and mass transfer along a wedge with heat source and concentration in the presence of suction or injection has been examined by Kandasamy et al. [9]. Govardhan et al. [10] presented a theoretical study on the influence of radiation on a steady free convection heat and mass transfer over an isothermal stretching sheet in the presence of a uniform magnetic field with viscous dissipation effect. Sattar [11] analyzed the effect of free and forced convection boundary layer flow through a porous medium with large suction. Similarly, Mohammed et al. [12] investigated the effect of similarity solution for MHD flow through vertical porous plate with suction. Jai [13] presented the study of a viscous dissipation and chemical reaction effects on flow past a stretching porous surface in a porous medium. In another article, a detailed numerical study on the combined effects of radiation and mass transfer on a steady MHD two-dimensional marangoni convection flow over a flat surface in presence of joule-heating and viscous dissipation under influence of suction and injection is studied by Ibrahim [14]. Khaleque and Samad [15] described the effects of radiation, heat generation, and viscous dissipation on MHD free convection flow along a stretching sheet.

When heat and mass transfer occur simultaneously in a moving fluid affecting each other causes a cross diffusion effect, the mass transfer caused by temperature gradient is called the Soret effect, while the heat transfer caused by concentration effect is called the Dufour effect. Soret and Dufour effects are important phenomena in areas such as hydrology, petrology, and geosciences. The Soret effect, for instance, has been utilized for isotope separation and in a mixture between gases with very light molecular weight (He, H_2_) and of medium molecular weight (N_2_, air). The Dufour effect was recently found to be of order of considerable magnitude so that it cannot be neglected, Eckert and Drake [16]. Many researchers studied Soret and Dufour effects; for example, Postelnicu [17] analyzed the effect of Soret and Dufour on heat and mass transfer. Chamkha and El-Kabeir [18] presented a theoretical study of Soret and Dufour effects on unsteady coupled heat and mass transfer by mixed convection flow over a vertical cone rotating in an ambient fluid in the presence of a magnetic field and chemical reaction. Usman and Uwanta [19] have considered the effect of thermal conductivity on MHD heat and mass transfer flow past an infinite vertical plate with Soret and Dufour effects. Similarly, Uwanta et al. [20] have analyzed MHD fluid flow over a vertical plate with Dufour and Soret effects. The effects of Soret and Dufour on an unsteady MHD free convection flow past a vertical porous plate in the presence of suction or injection have been investigated by Sarada and Shankar [21]. A numerical approach has been carried out for the study of Soret and Dufour effects on mixed convection heat and mass transfer past a vertical heated plate with variable fluid properties by Nalinakshi et al. [22]. Subhakar and Gangadhar [23] investigated the combined effects of the free convective heat and mass transfer on the unsteady two-dimensional boundary layer flow over a stretching vertical plate in the presence of heat generation/absorption and Soret and Dufour effects. In another article, Srinivasacharya and Reddy [24] examined Soret and Dufour effects on mixed convection in a non-Darcy porous medium saturated with micro polar fluid. Additionally, Sivaraman et al. [25] considered Soret and Dufour effects on MHD free convective heat and mass transfer with thermopheresis and chemical reaction over a porous stretching surface. Recently, Srinivasacharya and Upendar [26] analyzed the flow and heat and mass transfer characteristics of the mixed convection on a vertical plate in a micropolar fluid in the presence of Soret and Dufour effects. Most recently, a boundary layer analysis has been presented to study heat and mass transfer in the laminar, viscous, and incompressible fluid past a continuously moving plate saturated in a non-Darcy porous medium in the presence of Soret and Dufour effects with temperature dependent viscosity and thermal conductivity by El-Kabeir et al. [27]. Finally, Olanrewaju et al. [28] have investigated Dufour and Soret effects on convection heat and mass transfer in an electrically conducting power law flow over a heated porous plate.

In view of the above studies, the purpose of current investigation is to examine the influence of Soret and Dufour effects on MHD free convective heat and mass transfer flow over a vertical channel with constant suction and viscous dissipation.

## 2. Mathematical Formulation

Consider the flow of an unsteady laminar coupled free convective heat and mass transfer of an incompressible fluid past a vertical channel in a porous medium under the influence of a uniform transverse magnetic field and constant suction with viscous dissipation in the presence of Soret and Dufour effects. The *x*′-axis is taken on the finite plate and parallel to the free stream velocity which is vertical and the *y*′-axis is taken normal to the plate. All fluid properties are assumed to be constant. The magnetic field of small intensity is induced along the *y* direction. The fluid is assumed to be slightly conducting; hence, the magnetic Reynolds number is much less than unity and therefore the induced magnetic field is neglected in comparison with the applied magnetic field. Under the above assumptions, the general equations governing the flow can be expressed as follows:

(1) $$\begin{array}{c} \frac{\partial v^{\prime }}{\partial y^{\prime }}=0, \end{array}$$

(2) ∂u′∂t′−v0∂u′∂y′=v∂2u′∂y′2−σB02ρu′−vk∗u′+gβ(T′−T0′)+gβ∗(C′−C0′),

(3) ∂T′∂t′−v0∂T′∂y′=1ρCp∂∂y′[K(T)∂T′∂y′]+DMρCp∂2C′∂y′2+b∗u′2+1ρCp(∂u′∂y′)2,

(4) $$\begin{array}{c} \frac{\partial C^{\prime }}{\partial t^{\prime }}-v_{0}\frac{\partial C^{\prime }}{\partial y^{\prime }}=D\frac{\partial^{2}C^{\prime }}{\partial y^{\prime 2}}+D_{T}\frac{\partial^{2}T^{\prime }}{\partial y^{\prime 2}}, \end{array}$$

(5) $$\begin{array}{c} K\left(T\right)=k_{0}\left[1+\alpha \left(T_{w}^{\prime }-T\right)\right]. \end{array}$$

The corresponding initial and boundary conditions are prescribed as follows:

(6) $$\begin{array}{c} t\leq 0,\;u^{\prime }=0,\;T^{\prime }>T_{0},\;C^{\prime }>C_{0}\;\forall y^{\prime },\\ t>0,\;u^{\prime }=0,\;T^{\prime }=T_{w}^{\prime },\;C^{\prime }=C_{w}^{\prime }\;\text{at}\;\;y^{\prime }=0,\\ u^{\prime }=0,\;T^{\prime }=T_{0},\;C^{\prime }=C_{0}\;\text{at}\;\;y^{\prime }=H. \end{array}$$

**Figure 1:**
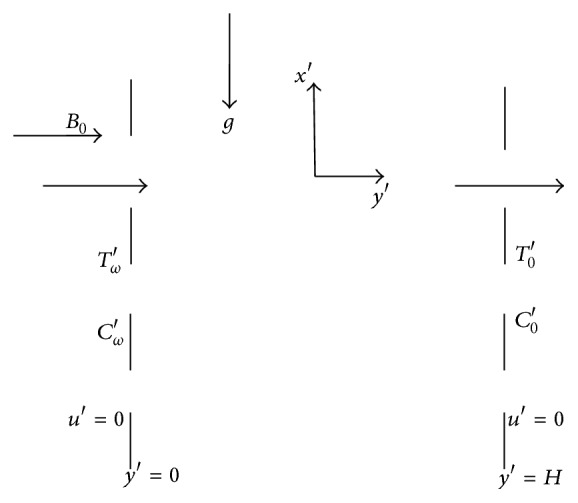
Geometry of the problem.

The geometry of the problem is shown in Figure 1.

From continuity equation, it is clear that the suction velocity is either a constant or a function of time. Hence, on integrating (1), the suction velocity normal to the plate is assumed in the form

(7) $$\begin{array}{c} v^{\prime }=-v_{0}, \end{array}$$

where *v*_0_  is a scale of suction velocity which is nonzero positive constant. The negative sign indicates that the suction is towards the plate and *v*_0_ > 0 corresponds to steady suction velocity normal at the surface. The fourth and fifth terms on the right hand side of (2) denote the thermal and concentration buoyancy effects, respectively, *u*′ and *v*′ are the velocity components in the *x*- and *y*-directions, respectively, *t* is the time,  *ν* is the kinematic viscosity, *g* is the acceleration due to gravity, *β* is the coefficient of volume expansion, *ρ* is the density, *β* ^*^ is the volumetric coefficient of expansion with concentration, *K*(*T*) is the thermal conductivity, *C*_*p*_ is the specific heat capacity at constant pressure, *k* ^*^ is the permeability of the porous medium, *D*_*M*_ is the coefficient of mass diffusivity, *k*_0_ is the thermal conductivity of the ambient fluid, *α* is a constant depending on the nature of the fluid, *D* is the coefficient of molecular diffusivity, *D*_*T*_ is the coefficient of temperature diffusivity, *b* ^*^ is the dimensionless joule-heating parameter, *σ* is the electric conductivity, and *B*_0_ is the magnetic field of constant strength. *T*′ and *T*_0_′ are the temperature of the fluid inside the thermal boundary layer and the fluid temperature in the free stream, respectively, *C*′ and *C*_0_′ are the corresponding concentrations.

On introducing the following nondimensional quantities:

(8) $$\begin{array}{c} u=\frac{u^{\prime }}{u_{0}},\;\;t=\frac{t^{\prime }u_{0}}{H},\;\;y=\frac{y^{\prime }}{H},\\ \theta =\frac{T^{\prime }-T_{0}^{\prime }}{T_{w}^{\prime }-T_{0}^{\prime }},\;\;C=\frac{C^{\prime }-{C^{\prime }}_{0}}{C_{w}^{\prime }-C_{0}^{\prime }},\\ \text{Pr}=\frac{u_{0}\rho C_{p}}{k_{0}},\;\;\text{Sc}=\frac{u_{0}}{D},\\ k=\frac{k^{*}u_{0}}{\nu H},\;\;M=\frac{\sigma B_{0}^{2}H}{\rho u_{0}},\\ \text{Gr}=\frac{Hg\beta \left(T_{w}^{\prime }-T_{0}^{\prime }\right)}{u_{0}^{2}},\;\;\text{Gc}=\frac{Hg\beta^{*}\left(C_{w}^{\prime }-C_{0}^{\prime }\right)}{u_{0}^{2}},\\ \;\;\lambda =\alpha \left(T_{w}^{\prime }-T_{0}^{\prime }\right),\;\;\gamma =\frac{\nu_{0}H}{u_{0}},\\ \text{Sr}=\frac{D_{T}\left(T_{w}^{\prime }-T_{0}^{\prime }\right)}{u_{0}\left(C_{w}^{\prime }-C_{0}^{\prime }\right)},\;\;\text{Ec}=\frac{H}{u_{0}\rho C_{p}\left(T_{w}^{\prime }-T_{0}^{\prime }\right)},\\ \text{Du}=\frac{D_{M}\left(C_{w}^{\prime }-C_{0}^{\prime }\right)}{u_{0}\rho C_{p}\left(T_{w}^{\prime }-T_{0}^{\prime }\right)},\;\;b_{1}=\frac{b^{*}H}{u_{0}\left(T_{w}^{\prime }-T_{0}^{\prime }\right)}. \end{array}$$

Applying (8), the set of (2), (3), (4), (5), and (6) reduces to the following:

(9) ∂u∂t−γ∂u∂y=∂2u∂y2−Mu−1ku+Grθ+GcC,∂θ∂t−γ∂θ∂y=λPr(∂θ∂y)2+1Pr(1+λθ)∂2θ∂y2+Du∂2C∂y2+b1u2+Ec(∂u∂y)2,∂C∂t−γ∂C∂y=1Sc∂2C∂y2+Sr∂2θ∂y2

with the following initial and boundary conditions:

(10) $$\begin{array}{c} t\leq 0,\;u=0,\;\theta =0,\;C=0,\;\forall y,\\ t>0,\;u=0,\;\theta =1,\;C=1,\;\text{at}\;y=0,\\ u=0,\;\theta =0,\;C=0\;\text{at}\;\;y=1, \end{array}$$

where Ec is the Eckert number, Pr is the Prandtl number, Sc is the Schmidt number, Sr is the Soret number, Du is the Dufour number, *M* is the Magnetic field parameter, Gr is the thermal Grashof number, Gc is the Solutal Grashof number, *k*  is the porous parameter,  *b*_1_ is the joule-heating parameter, *λ* is the variable thermal conductivity, and *γ* is the variable suction parameter while *u* and *v* are dimensionless velocity components in *x*- and *y*-directions, respectively, and *t* is the dimensionless time.

The skin friction, Nusselt number, and Sherwood number are important physical parameters for this type of boundary layer flow and are given by

(11) $$\begin{array}{c} C_{f}={\left(\frac{\partial u}{\partial y}\right)}_{y=0},\;\;\text{Nu}=-{\left(\frac{\partial \theta }{\partial y}\right)}_{y=0},\;\;\text{Sh}={\left(\frac{\partial C}{\partial y}\right)}_{y=0}. \end{array}$$

## 3. Numerical Solution Procedure

The set of coupled nonlinear governing boundary layer equation (9) together with boundary conditions (10) are solved numerically by using the implicit finite difference method of Crank-Nicolson type. The finite difference approximations equivalent to (9) are as follows:

(12) (uij+1−uijΔt)−γ2Δy(ui+1j−ui−1j) =12(Δy)2(ui+1j+1−2uij+1+ui−1j+1+ui+1j−2uij+ui−1j)  −(M+1k)uij+Gr(θ)ij+Gc(C)ij,(θij+1−θijΔt)−γ2Δy(θi+1j−θi−1j) =H2Pr(Δy)2(θi+1j+1−2θij+1+θi−1j+1+θi+1j−2θij+θi−1j)  +Du2(Δy)2(Ci+1j+1−2Cij+1+Ci−1j+1+Ci+1j−2Cij+Ci−1j)  +λPr(Δy)2(θi+1j−θij)2  +Ec(Δy)2(ui+1j−uij)2+b1(uij)2(Cij+1−CijΔt)−γ2Δy(Ci+1j−Ci−1j)  =12Sc(Δy)2   ×(Ci+1j+1−2Cij+1+Ci−1j+1+Ci+1j−2Cij+Ci−1j)   +Sr2(Δy)2(θi+1j+1−2θij+1+θi−1j+1+θi+1j−2θij+θi−1j).

The initial and boundary conditions take the following forms:

(13) $$\begin{array}{c} u_{i,j}=0,\;\;\theta_{i,j}=0,\;\;C_{i,j}=0\\ u_{0,j}=0,\;\;\theta_{0,j}=1,\;\;C_{0,j}=1\\ u_{H,j}=0,\;\;\theta_{H,j}=0,\;\;C_{H,j}=0, \end{array}$$

where *H* corresponds to 1.

Equations (12) are simplified as follows:

(14) −U1ui−1j+1+U2uij+1−U1ui+1j+1  =U3ui−1j+U4uij+U5ui+1j+U6θij+U7Cij,

(15) −T1θi−1j+1+T2θij+1−T1θi+1j+1  =T3θi−1j+T4θij+T5θi+1j+T6(θi+1j−θij)2   +T7(ui+1j−uij)2+T8(uij)2   +T9(Ci+1j+1−2Cij+1+Ci−1j+1+Ci+1j−2Cij+Ci−1j),

(16) −V1Ci−1j+1+V2Cij+1−V1Ci+1j+1  =V3Ci−1j+V4Cij+V5Ci+1j   +V6(θi+1j+1−2θij+1+θi−1j+1+θi+1j−2θij+θi−1j).

The index *i* corresponds to space *y* and *j* corresponds to time *t*. Δ*y* and Δ*t* are the mesh sizes along *y*-direction and time *t*-direction, respectively. The finite difference equations (14)–(16) at every internal nodal point on a particular *n*-level constitute a tridiagonal system of equations, which are solved by using Thomas algorithm. During the computations in each time step, the coefficients in (14)–(16) are treated as constants. The values of *θ*, *C*, and *u* are known at all grid points at the initial time *t* = 0. The values of *θ*, *C*, and *u* at time level (*j* + 1) using the known values at previous time level *j* are calculated as follows.

Knowing the values of *θ*, *C*, and *u* at a time *t* = *j*, calculate *θ* and *C* at time *t* = *j* + 1 using the finite difference equations (15) and (16) and solving the tridiagonal system of equations by using Thomas algorithm as discussed by Carnahan et al. [29]. Knowing the values of *θ* and  *C* at time *t* = *j* and *t* = *j* + 1 and the values of *u* at time *t* = *j*, solve (14) using tridiagonal matrix inversion, to obtain the values of *u* at time *t* = *j* + 1. This process is repeated for various *i* levels. Thus the values of *θ*, *C*  , and *u* are known at all grid points in the rectangular region at (*j* + 1)th time level. Computations are carried out until the steady state is reached. The Implicit Crank-Nicolson method is a second order method *O*(Δ*t*)^2^ in time and has no restrictions on space Δ*y* and time step Δ*t*; that is, the method is compatible. Hence the finite difference scheme is unconditionally stable and therefore compatibility and stability ensures the convergence of the scheme. Computations are carried out for different values of physical parameters involved in the problem.

## 4. Results and Discussion

**Figure 2:**
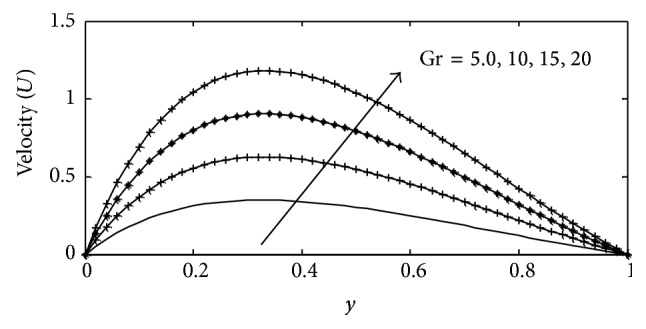
Velocity profile for different values of Gr  with Gc = 2, *γ* = 3, Sc = 0.22, *b*_1_ = 0.01, *λ* = 0.5, *M* = 1, *k* = 1, Sr = 1, Du = 0.1, Ec = 0.01, Pr = 0.71, and *t* = 02.

**Figure 3:**
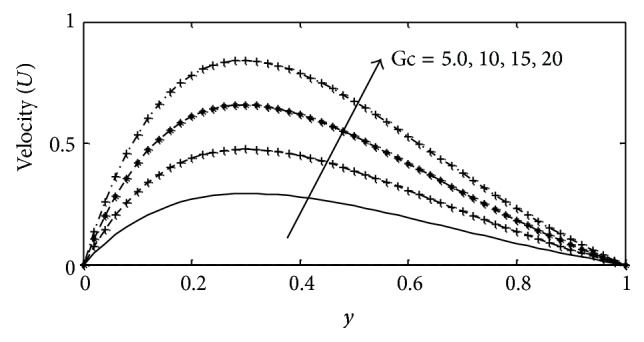
Velocity profile for different values of Gc with Gr = 2, *γ* = 3, Sc = 0.22, *b*_1_ = 0.01, *λ* = 0.5,  *M* = 1, *k* = 1, Sr = 1, Du = 0.1, Ec = 0.01, Pr = 0.71, and *t* = 0.2.

**Figure 4:**
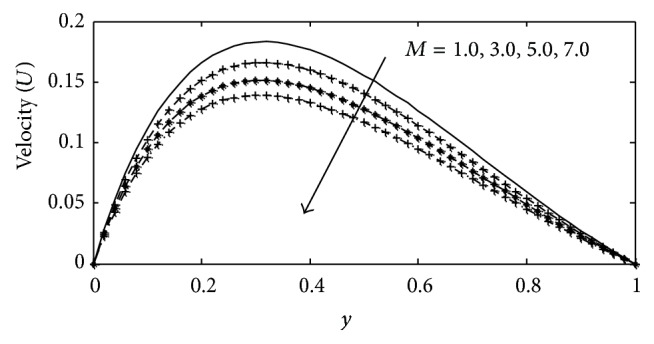
Velocity profile for different values of *M* with Gr = 2, Gc = 2, *γ* = 3, Sc = 0.22, *b*_1_ = 0.01, *λ* = 0.5, *k* = 1, Sr = 1, Du = 0.1, Ec = 0.01, Pr = 0.71, and *t* = 0.2.

**Figure 5:**
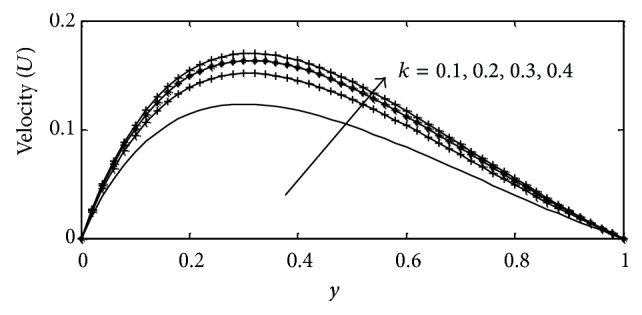
Velocity profile for different values of *k* with Gr = 2, Gc = 2, *γ* = 3, Sc = 0.22, *b*_1_ = 0.01, *λ* = 0.5, *M* = 1, Sr = 1, Du = 0.1, Ec = 0.01, Pr = 0.71, and *t* = 0.2.

In this paper, numerical values are assigned physically to the embedded parameters in the system in order to report on the analysis of the fluid flow structure with respect to velocity, temperature, and concentration profiles. Numerical results for velocity, temperature, and concentration profiles are presented on graphs, while the skin-friction coefficient, Nusselt number, and Sherwood number are shown in tabular form. The Prandtl number is taken to be (Pr = 0.71,7.0) which corresponds to air and water. The value of Schmidt number is taken as (Sc = 0.22,0.66,0.94,2.62) representing diffusing chemical species of most common interest in air for hydrogen, oxygen, carbon dioxide, and propyl benzene, while for Soret numbers (2.50) and (6.89) corresponding to thermal diffusion ratio of (H_2_–CO_2_) and (He–Ar) are chosen, respectively, while other parameters in the flow are chosen arbitrarily. The influence of thermal Grashof number Gr  and solutal Grashof number Gc on the velocity is presented in Figures 2 and 3. The thermal Grashof number signifies the relative effect of the thermal buoyancy force to the viscous hydrodynamic force in the boundary layer, while the solutal Grashof number defines the ratio of the species buoyancy force to the viscous hydrodynamic force. As expected the fluid velocity increases due to the enhancement of thermal and species buoyancy forces. The velocity distribution increases rapidly near the porous plate and then decreases smoothly to the free stream value. For different values of magnetic parameter *M* and porous parameter *k*, the velocity profiles are plotted in Figures 4 and 5, respectively. It can be seen that as *M* increases, the velocity decreases. This results agrees with the expectations since the magnetic field exerts a restraining force on the fluid which tends to impede it is motion.  Figure 5 depicts the effect of the porous parameter *k* on the velocity profile. An increase in *k* increases the resistance of the porous medium, which will tend to accelerate the flow and therefore increase the velocity.

**Figure 6:**
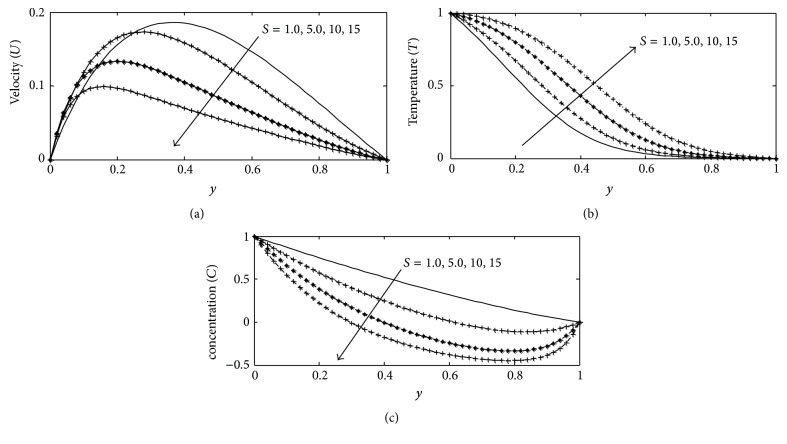
(a) Velocity profile for different values of (*γ* = *S*) with Gr = 2, Gc = 2, *k* = 1, Sc = 0.22, *b*_1_ = 0.01, *λ* = 0.5, *M* = 1, Sr = 1, Du = 0.1, Ec = 0.01, Pr = 0.71, and *t* = 0.2. (b) Temperature profile for different values of (*γ* = *S*) with Gr = 5, Gc = 2, Sc = 0.22, *b*_1_ = 0.1, *λ* = 1, *M* = 1, *k* = 1, Sr = 0.2, Du = 0.2, Ec = 0.01, Pr = 0.71, and *t* = 0.02. (c) Concentration profile for different values of (*γ* = *S*) with Gr = 2, Gc = 2, Sc = 0.22, *b*_1_ = 0.01, *λ* = 0.5, *M* = 1, *k* = 1, Sr = 3, Du = 0.1, Ec = 0.01, Pr = 0.71, and *t* = 0.2.

**Figure 7:**
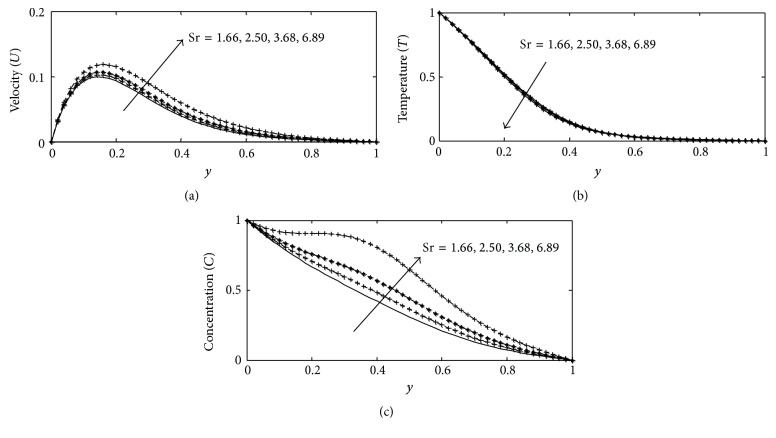
(a) Velocity profile for different values of Sr with Gr = 2, Gc = 2, *γ* = 3, Sc = 0.22, *b*_1_ = 0.01, *λ* = 0.5, *k* = 1, *M* = 1, Du = 0.1, Ec = 0.01, Pr = 0.71, and *t* = 0.2. (b) Temperature profile for different values of Sr with Gr = 5, Gc = 2, *γ* = 0.1, Sc = 0.22, *b*_1_ = 0.1, *λ* = 1, *k* = 1, *M* = 1, Du = 0.2, Ec = 0.01, Pr = 0.71, and *t* = 0.02. (c) Concentration profile for different values of Sr with Gr = 2, Gc = 2, Sc = 0.22, *b*_1_ = 0.01, *λ* = 0.5, *M* = 1, *k* = 1, *γ* = 3, Du = 0.1, Ec = 0.01, Pr = 0.71, and *t* = 0.02.

**Figure 8:**
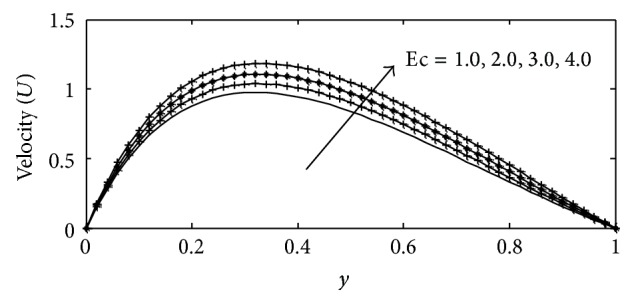
Velocity profile for different values of Ec with Gr = 2, Gc = 2, *γ* = 3, Sc = 0.22, *b*_1_ = 0.01, *λ* = 0.5, *k* = 1, Sr = 1, Du = 0.1, *M* = 1, Pr = 0.71, and *t* = 0.2.

**Figure 9:**
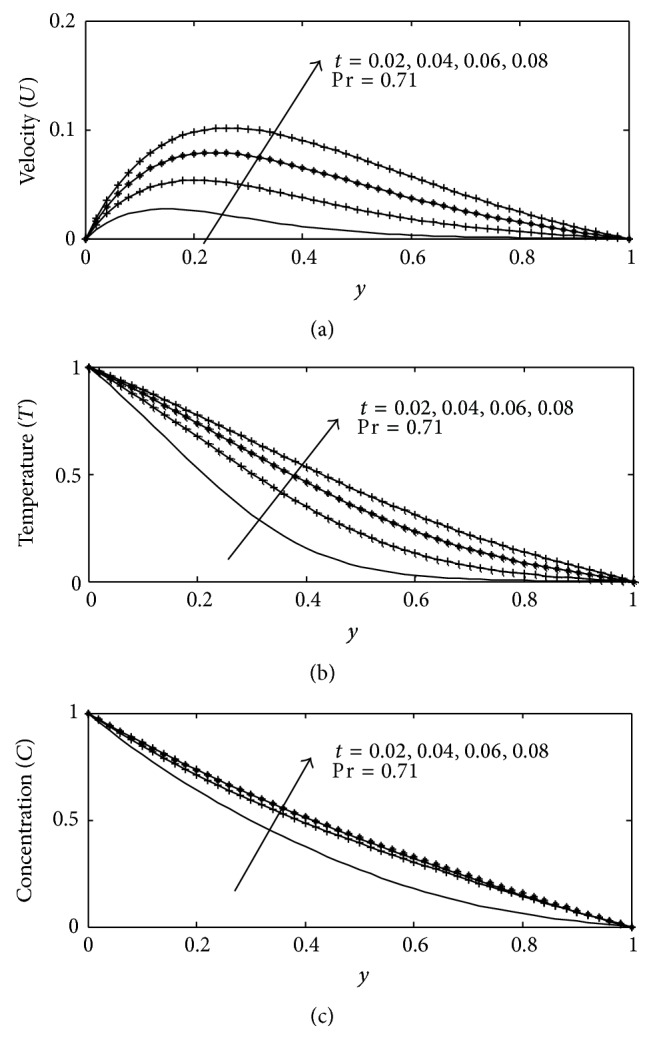
(a) Velocity profile for different values of *t* with Gr = 2, Gc = 2, *γ* = 3, Sc = 0.22, *b*_1_ = 0.01, *λ* = 0.5, *k* = 1,  Sr = 1, Du = 0.1, Ec = 0.01, and *M* = 1. (b) Temperature profile for different values of *t* with Gr = 5, Gc = 2, Sc = 0.22, *b*_1_ = 0.1, *λ* = 1, *M* = 1, *k* = 1, Sr = 0.2, Du = 0.2, Ec = 0.01, and *γ* = 0.1. (c) Concentration profile for different values of *t* with Gr = 2, Gc = 2, Sc = 0.22, *b*_1_ = 0.01, *λ* = 0.5, *M* = 1, *k* = 1, Sr = 1, Du = 0.1, Ec = 0.01, and *γ* = 0.1.

**Figure 10:**
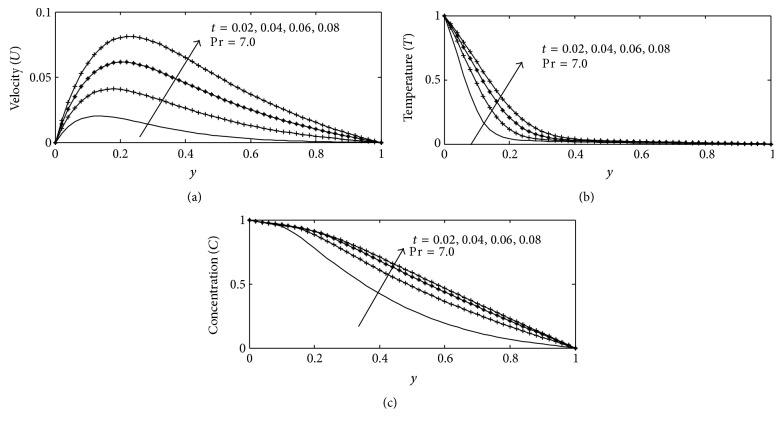
(a) Velocity profile for different values of *t* with Gr = 2, Gc = 2, *γ* = 3, Sc = 0.22, *b*_1_ = 0.01, *λ* = 0.5, *k* = 1, Sr = 1, Du = 0.1, Ec = 0.01, and *M* = 1. (b) Temperature profile for different values of *t* with Gr = 5, Gc = 2, Sc = 0.22, *b*_1_ = 0.1, *λ* = 1, *M* = 1, *k* = 1, Sr = 0.2, Du = 0.2, Ec = 0.01, and *γ* = 0.1. (c) Concentration profile for different values of *t* with Gr = 2, Gc = 2, Sc = 0.22, *b*_1_ = 0.01, *λ* = 0.5, *M* = 1, *k* = 1, Sr = 1, Du = 0.1, Ec = 0.01, and *γ* = 0.1.

For various values of suction parameter *γ*, the velocity, temperature, and concentration profiles are plotted in Figures 6(a)–6(c). It is found out that an increase in the suction parameter causes a fall in the velocity and concentration profiles throughout the boundary layer, while increase in the temperature profiles. This is due to the fact that suction parameter stabilizes the boundary layer growth. The effect of Soret number Sr on velocity, temperature, and concentration profiles is illustrated in Figures 7(a)–7(c) respectively. The Soret number defines the effect of the temperature gradients inducing significant mass diffusion effects. It can be seen that the velocity and concentration profiles increase with an increase in Sr, while a rise in Sr causes a fall in the temperature profiles within the boundary layer. These behaviors are evident from Figures 7(a)–7(c). The influence of viscous dissipation parameter, that is, Eckert number Ec, on the velocity profiles is depicted in Figure 8. The Eckert number expresses the relationship between the kinetic energy in the flow and the enthalpy. It embodies the conversion of kinetic energy into internal energy by work done against the viscous fluid stress. It is observed that greater viscous dissipative heat causes an increase in the velocity profiles across the boundary layer. Figures 9(a)–10(c) describe the behavior for velocity, temperature, and concentration profiles for different values of nondimensional time *t* for two working fluids air (Pr = 0.71) and water (Pr = 7.0). It is evident from these figures that as time increases the velocity, temperature, and concentration increase. The consequence of temperature increase results from increase in time since convection current becomes stronger and hence velocity and concentration increase with time.

**Figure 11:**
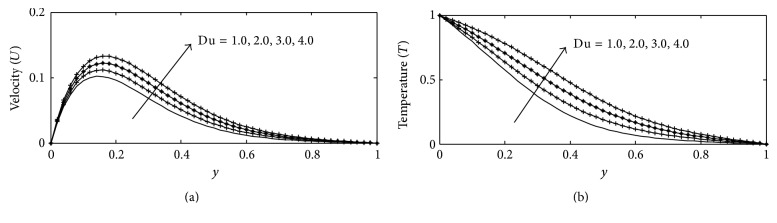
(a) Velocity profile for different values of Du with Gr = 2, Gc = 2, *γ* = 3, Sc = 0.22, *b*_1_ = 0.01, *λ* = 0.5, *k* = 1, Sr = 1, *M* = 1, Ec = 0.01, Pr = 0.71, and *t* = 0.2. (b) Temperature profile for different values of Du with Gr = 5, Gc = 2, Sc = 0.22, *b*_1_ = 0.1, *λ* = 1, *M* = 1, *k* = 1, Sr = 0.2, *γ* = 0.1, Ec = 0.01, Pr = 0.71, and *t* = 0.02.

**Figure 12:**
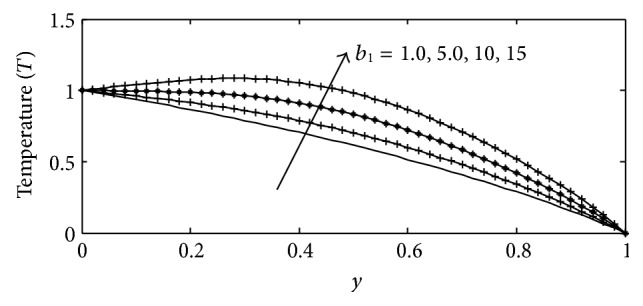
Temperature profile for different values of *b*_1_ with Gr = 5, Gc = 2, Sc = 0.22, *γ* = 0.1, *λ* = 1, *M* = 1, *k* = 1, Sr = 0.2, Du = 0.2, Ec = 0.01, Pr = 0.71, and *t* = 0.2.

**Figure 13:**
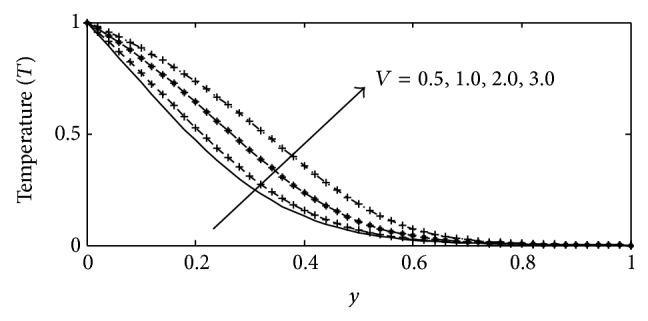
Temperature profile for different values of (*λ* = *v*) with Gr = 5, Gc = 2, Sc = 0.22, *b*_1_ = 0.1, *γ* = 0.1, *M* = 1, *k* = 1, Sr = 0.2, Du = 0.2, Ec = 0.01, Pr = 0.71, and *t* = 0.02.

**Figure 14:**
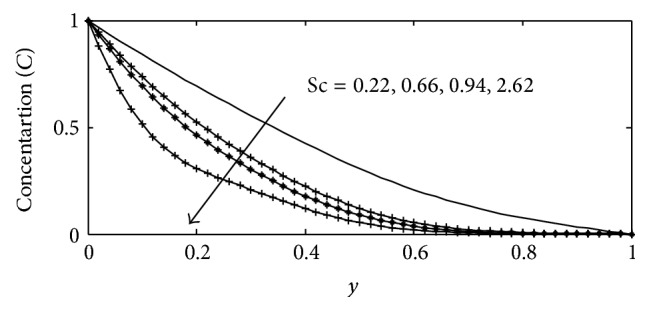
Concentration profile for different values of Sc with Gr = 2, Gc = 2, *γ* = 0.1, *b*_1_ = 0.01, *λ* = 0.5, *M* = 1, *k* = 1, Sr = 1, Du = 0.1, Ec = 0.01, Pr = 0.71, and *t* = 0.02.

Figures 11(a) and 11(b) graphically show the influence of Dufour number Du on the velocity and temperature profiles, respectively. The Dufour number signifies the contribution of the concentration gradients to the thermal energy flux in the flow. It is seen that as Du increases there is monotonic increase in the velocity and temperature profiles. For different values of joule-heating parameter *b*_1_ and thermal conductivity parameter *λ*, the temperature profiles are plotted in Figures 12 and 13, respectively. It is observed that increasing the joule-heating parameter and thermal conductivity parameter produces significant increase in the thermal conduction of the fluid, which is physically true because as the thermal conductivity increases the temperature within the fluid increases.  Figure 14 describes the behavior of various values of Scmidt number Sc on the concentration profiles. The Schmidt number characterizes the ratio of thicknesses of viscous to the mass diffusivity. The Scmidt number quantifies the relative effectiveness of momentum and mass transport by diffusion in the velocity and concentration boundary layers. It is observed that increase in the values of Sc causes the species concentration and its boundary layer thickness to decrease significantly.

The effects of various governing parameters on skin-friction coefficient *C*_*f*_, Nusselt number Nu, and Sherwood number Sh are shown in Tables 1 and 2. In order to highlight the contributions of each parameter, one parameter is varied while the rest take default fixed values. It is observed from Table 1 that an increase in any of the parameters *M* and *γ* causes reduction in the skin-friction, while increasing any of the parameters, Gr, Gc, *k*, and *λ* resulted in corresponding increase in the skin-friction coefficient. It is also seen that as *λ* and *γ* increase, there is a rise in Nusselt number and Sherwood number, respectively. From Table 2, it is observed that an increase in Ec, *b*_1_, and Du leads to a rise in the skin-friction coefficient, Nusselt number, and Sherwood number, respectively, while an increase in Pr leads to a fall in the skin-friction coefficient, Nusselt number, and Sherwood number, respectively. It is also seen that as Sr and Sc increase there is a fall in the skin-friction coefficient and a rise in Nusselt and Sherwood numbers, respectively.

## 5. Conclusions

The present paper analyzes the influence of Soret and Dufour effects on MHD free convective heat and mass transfer flow over a vertical channel with constant suction and viscous dissipation. The resulting partial differential equations are nondimensionalised, simplified, and solved by implicit finite difference method of Crank-Nicolson type. From the present numerical study the following conclusions can be drawn.

1. Velocity profiles increased due to increase in thermal Grashof number, solutal Grashof number, porous parameter, Eckert number, Soret number, Dufour number, and dimensionless time while it decreased due to increase in magnetic parameter and suction parameter.
2. An increase in temperature profiles is a function of an increase in thermal conductivity, suction parameter, Eckert number, joule-heating parameter, Dufour number, and dimensionless time while it decreased due to increase in Soret number.
3. Concentration profiles decreased due to increases in Schmidt number and suction parameter while it increased due to increase in Soret number and dimensionless time.
4. There is a rise in the skin-friction coefficient, Nusselt number, and Sherwood number due to increase in Eckert number, thermal conductivity parameter, joule-heating parameter, and Dufour number while a fall is observed in skin-friction coefficient with increase in Soret number and Prandtl number.

## Figures and Tables

**Table 1:** Values of skin-friction coefficient *C*_*f*_, Nusselt number Nu, and Sherwood number Sh for various values of physical parameters (Gr, Gc, *M*, *k*, λ, and γ).

Gr	Gc	*M*	*k*	λ	γ	*C*_*f*_	Nu	Sh
2.0	2.0	1.0	0.5	1.0	2	0.2611	3.3328	0.6171
**4.0**	2.0	1.0	0.5	1.0	2	0.4720	3.3451	0.6221
2.0	**4.0**	1.0	0.5	1.0	2	0.3119	3.3386	0.6195
2.0	2.0	**3.0**	0.5	1.0	2	0.2353	3.3317	0.6167
2.0	2.0	1.0	**1.0**	1.0	2	0.2757	3.3334	0.6174
2.0	2.0	1.0	0.5	**2.0**	2	0.2897	8.1676	3.4334
2.0	2.0	1.0	0.5	1.0	**3.0**	0.2072	4.5600	1.3689

With fixed values of (Pr = 0.71, Ec = 0.01, Sc = 0.6, Du = 0.2, *b*_1_ = 1.0, Sr = 1.0, and *t* = 0.2).

**Table 2:** Values of skin-friction coefficient *C*_*f*_, Nusselt number Nu, and Sherwood number Sh for various physical parameters (Pr, *b*_1_, Ec, Sr, Du, and Sc).

Pr	*b*_1_	Ec	Sr	Du	Sc	*C*_*f*_	Nu	Sh
0.71	1.0	2.0	1.0	0.2	0.22	0.4038	2.0257	1.4344
**3.0**	1.0	2.0	1.0	0.2	0.22	0.2898	0.6168	1.4296
0.71	**2.0**	2.0	1.0	0.2	0.22	0.4041	2.0311	1.4354
0.71	1.0	**3.0**	1.0	0.2	0.22	0.4053	2.0729	1.4713
0.71	1.0	2.0	**1.66**	0.2	0.22	0.4019	2.0523	1.5730
0.71	1.0	2.0	1.0	**1.0**	0.22	0.4133	3.2477	1.6418
0.71	1.0	2.0	1.0	0.2	**0.94**	0.3519	2.0983	1.7267

With fixed values of (Gr = 2.0, Gc = 2.0, *M* = 1.0, γ = 0.5, λ = 1.0, *k* = 0.5, and *t* = 0.2).

## Nomenclature

*C*: Concentration
*C*_*p*_: Specific heat at constant pressure
*D*: Mass diffusivity
*D*_*M*_: Coefficient of Mass diffusivity
*D*_*T*_: Coefficient of temperature diffusivity
Du: Dufour number
*g*: Acceleration due to gravity
Gr: Grashof number
Gc: Solutal Grashof number
*k*: Permeability of the porous medium
Nu: Nusselt number
Pr: Prandtl number
Sc: Schmidt number
Sr: Soret number
*T*: Temperature
*u*, *v*: Velocities in the *x*- and *y*-direction, respectively
*x*, *y*: Cartesian coordinates along the plate and normal to it, respectively
*B*_0_: Magnetic field of constant strength
Ec: Eckert number
*M*: Magnetic field parameter
*b*_1_: Joule-heating parameter.

### Greek Letters

*β*^*^: Coefficient of expansion with concentration
*β*: Coefficient of thermal expansion
*ρ*: Density of fluid
*σ*: Electric conductivity
*θ*: Dimensionless temperature
*ν*: Kinematic viscosity
*γ*: Suction parameter
*λ*: Variable thermal conductivity.

### Subscripts

*w*: Condition at wall.
